# Genomic Features of Antimicrobial Resistance and Virulence in Multidrug-Resistant *Vibrio furnissii*

**DOI:** 10.3390/vetsci12121180

**Published:** 2025-12-10

**Authors:** Xuemei Wu, Wenhui Zhang, Ming Liu, Zhiqiang Wang, Ruichao Li

**Affiliations:** 1 Jiangsu Co-Innovation Center for Prevention and Control of Important Animal Infectious Diseases and Zoonoses, College of Veterinary Medicine, Yangzhou University, Yangzhou 225009, China; wuxuemei63@outlook.com (X.W.); wenhuizhang_yzu@outlook.com (W.Z.); zqwang@yzu.edu.cn (Z.W.); 2 Institute of Comparative Medicine, Yangzhou University, Yangzhou 225009, China; 3 College of Nursing and Health Management, Wuhan Donghu College, Wuhan 430040, China; 4 National Key Laboratory of Agricultural Microbiology, Huazhong Agricultural University, Wuhan 430070, China

**Keywords:** pAQU, *bla*_GMA-1_, *zot*, *Vibrio furnissii*

## Abstract

*V. furnissii* is a marine bacterium capable of infecting both invertebrates and humans. However, the mechanisms underlying its drug resistance and virulence remain largely elusive. In this study, we isolated a multidrug-resistant *V. furnissii* strain, MT14, from bivalve mollusks. Genomic analysis revealed that MT14 carries a novel pAQU-type plasmid, designated pMT14, which harbors six antibiotic resistance genes: *qnrS2*, *qnrVC6*, *dfrA31*, *tetA*; *sul2*, and *bla*_GMA-1_. Notably, *bla*_GMA-1_ and its variants confer resistance to penicillin-class antibiotics, including ampicillin, amoxicillin, and carbenicillin. Comparative genomic analysis further revealed that *V*. *furnissii* strains commonly encode T6SS1, T6SS2, and the hemolysin gene *vfh*, while only five sequenced strains carry *zot* toxin gene. To our knowledge, this is the first report of *zot* in *V*. *furnissii*. In addition, the absence of drug resistance genes in the majority of genomes (73.3%, 22/30) suggests that such traits are not intrinsic to the species but have been acquired by specific strains, likely through plasmid transfer or other mobile genetic elements. The emergence of *bla*_GMA-1_ and *zot* in *V*. *furnissii* represents a potential public health concern, underscoring the need for enhanced surveillance of antimicrobial resistance in marine pathogens.

## Simple Summary

A multidrug-resistant *V. furnissii* strain MT14 was isolated from Manila clams. Its resistance genes were identified on a novel plasmid and a chromosomal transposon, including one conferring resistance to common penicillins. As most *V. furnissii* strains are not inherently drug-resistant, these traits were likely acquired. We also identified a toxin gene in *V. furnissii* that may enhance its virulence. Our findings highlight a potential public health risk and underscore the need for enhanced surveillance of antibiotic resistance in vibrios from filter-feeding shellfish.

## 1. Introduction

*Vibrio furnissii* is a zoonotic bacterium commonly found in marine environments, which can result in high mortality in farmed shellfish [1,2]. This pathogen, which is phylogenetically close to species such as *V. cholerae* and *V. fluvialis*, is also capable of infecting humans. Symptoms of infection include gastroenteritis and extraintestinal infections [3,4]. Genomic analyses have identified several putative virulence factors in *V. furnissii*, such as *ilpA*, *vfh*, *hupO*, T6SS, and *vfp* [5]. Experimental studies have confirmed the in vitro expressing and activity of a hemolysin (encoded by *vhf*) and the T6SS. However, due to strain-dependent variations in T6SS gene cluster composition [4,6], the specific genetic loci examined in these studies remain ambiguous, and the contributions of hemolysin and T6SS to pathogenicity are yet to be fully elucidated. Whether *V. furnissii* encodes other virulence factors is largely unknown.

pAQU-type plasmids, initially characterized in *Photobacterium damselae* subsp. *damselae*, *Photobacterium aphoticum*, and *V. splendidus*, have since been identified in other *Vibrio* species, including *V. harveyi*, *V. parahaemolyticus*, and *V. alginolyticus* [7,8,9]. Despite their expanding host range, the overall prevalence and genetic diversity of pAQU-type plasmids across the *Vibrio* genus remain inadequately characterized. These plasmids exhibit substantial heterogeneity in antibiotic resistance gene content. For instance, the *bla*_GMA-1_ is present in pAQU1 but absent in pVPH1 [8,10]. Functional characterization of *bla*_GMA-1_ demonstrated that, when expressed in *E. coli* under its native promoter, this gene confers resistance to ampicillin, carbenicillin, piperacillin, and the first-generation cephalosporin cefazolin, but not to later-generation cephalosporins, indicating its preferential hydrolysis of penicillins [10]. Notably, the use of chloramphenicol in oyster hatcheries has been shown to promote the dissemination of the pAQU-type plasmid, pAQU-MAN, among resident *Vibrio* populations [11]. This is of particular concern given that although penicillins have historically been used to treat *Vibrio* infections, their clinical efficacy is often limited [12]. The recent emergence of penicillin-resistant *V*. *furnissii* strains underscores the critical need for enhanced surveillance of antibiotic resistance and systematic investigation of virulence mechanisms in this pathogen [13,14].

Given that *Vibrio* species are frequently associated with seafood, we isolated a multidrug-resistant *V. furnissii* strain, MT14, from Manila clams. This strain carries a novel pAQU-type plasmid, pMT14, which harbors *bla*_GMA-1_, a gene known to confer resistance to penicillin-class antibiotics such as ampicillin, amoxicillin, and carbenicillin, a property shared with its variants *bla*_GMA-2_, *bla*_GMA-3_, and *bla*_GMA-4_. Additionally, we identified a transposition unit containing multiple resistance genes inserted into a *umuC*-like gene on chromosome I of MT14. Comparative genomic analysis revealed that while T6SS1 and T6SS2 are widespread among *V. furnissii* strains, the *zot* toxin gene is rare and was likely acquired through horizontal gene transfer. To our knowledge, this is the first report of *zot* in *V. furnissii*. Our findings demonstrate that *V. furnissii* can serve as a host for pAQU-type plasmids and highlight the emergence of *bla*_GMA-1_ and *zot* in this species, posing a potential public health threat. These results underscore the need for ongoing surveillance of antibiotic resistance and virulence gene dissemination in *Vibrio* pathogens.

## 2. Materials and Methods

### 2.1. Strain Isolation

In April 2021, approximately 250 g of *Ruditapes philippinarum* (Manila clam) was purchased from a supermarket in Nanjing city, China. The bivalve was homogenized and incubated in 100 mL of marine broth (Difco, Detroit, MI, USA) supplemented with 120 μg/mL amoxicillin at 30 °C overnight. Subsequently, 1 mL of the bacterial culture was serially diluted, and 100 μL aliquots were plated onto thiosulfate-citrate-bile salts-sucrose (TCBS) agar containing 120 μg/mL amoxicillin. After overnight incubation at 30 °C, one representative yellow colony was randomly selected for purification. One isolate, designated MT14, was obtained for further analysis.

### 2.2. Antimicrobial Susceptibility Testing and Conjugation Assay

The antimicrobial susceptibility testing was performed using the broth microdilution method in accordance with the CLSI M45 guideline. The testing conditions, including inoculum preparation, incubation time, and temperature, followed the recommendations outlined in the M45 document [15].

The conjugation assay was performed as described previously [14]. In brief, donor strain MT14 and the rifampicin-resistant recipient strain *E. coli* C600 were grown overnight, mixed in a 5:1 ratio, and pelleted by centrifugation. The cell pellet was resuspended in 50 μL fresh Luria–Bertani (LB) broth. The entire mixture was spotted onto a sterile membrane placed on the surface of an LB agar plate and incubated for 12–16 h at 37 °C. Following incubation, transconjugants were selected by plating on an LB agar plate supplemented with 300 µg/mL rifampicin and 100 µg/mL amoxicillin.

### 2.3. Whole-Genome Sequencing and Bioinformatics Analysis

Genomic DNA of strain MT14 was extracted, and then the DNA was sequenced using the Illumina (San Diego, CA, USA) short-read and Oxford Nanopore (Oxford, UK) MinION long-read platforms. Reads were assembled using Unicycler v0.4.8, and then RAST was used for genome annotation as we described previously [8,14]. For antibiotic resistance genes identification, ResFinder was used (https://genepi.food.dtu.dk/resfinder, accessed on 30 September 2025). For insertion sequence identification, ISFinder (https://www-is.biotoul.fr/, accessed on 30 September 2025) was used. For virulence gene prediction, VFDB (http://www.mgc.ac.cn/VFs/, accessed on 30 September 2025) was used. All available *V*. *furnissii* genome data were downloaded from the NCBI Genome database, and the phylogenetic tree of 30 *V*. *furnissii* strains was constructed using Roary and FastTree based on single nucleotide polymorphism (SNPs) of core genomes. The tree was visualized and annotated using iTOL v5 (https://itol.embl.de/itol.cgi, accessed on 30 September 2025).

## 3. Results

A screening of a market-sourced batch of Manila clams for amoxicillin-resistant vibrios resulted in the isolation of *Vibrio furnissii* strain MT14. Antimicrobial susceptibility testing showed that MT14 was resistant to ampicillin, amoxicillin, ciprofloxacin, chloramphenicol, kanamycin, streptomycin, and tetracycline, but remained susceptible to colistin, ceftriaxone, meropenem, and tigecycline (Table 1). A conjugation assay was performed to assess the transferability of this resistance phenotype. However, no transconjugants were obtained under the tested conditions, suggesting that the resistance genes were not transmissible under such conditions.

**Figure 1:**
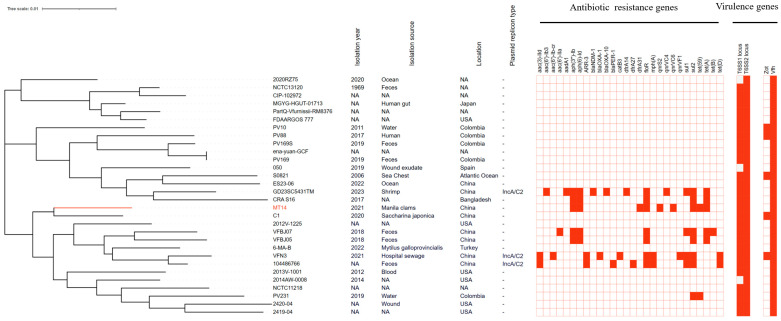
Genomic sequences analysis of 30 *V. furnissii* strains. Core-genome-based phylogenetic tree of the *V*. *furnissii* strains. Antimicrobial resistance and virulence factors are represented by red squares. The strains *V. furnissii* Colony82, Colony85, Colony603, and PV57 were excluded from the analysis due to low-quality genome sequences. NA, information not available.

To elucidate the multidrug resistance mechanism of *V*. *furnissii* MT14, we sequenced its complete genome. PubMLST analysis confirmed MT14 as *V*. *furnissii*, harboring two chromosomes and one plasmid, pMT14. The core-genome phylogeny of 30 *V*. *furnissii* strains revealed three distinct clades, with strain MT14 clustering closely with the *Saccharina japonica* isolate C1, implying a shared niche or common habitat (Figure 1).

Given the established roles of hemolysin, T6SS, and Zot in virulence for *V*. *cholerae* and *V*. *fluvialis* [16,17,18,19,20], we hypothesized these factors may similarly contribute to the pathogenicity of *V. furnissii*. Using VFDB, we analyzed the distribution of *vfh*, *zot*, and T6SSs loci across 30 *V*. *furnissii* genomes. The results showed that 27 strains encoded both T6SS1 and T6SS2, while strains 2014AW-0008, 2020RZ75, and 050 lacked T6SS1. Given the phylogenetic proximity of *V*. *furnissii* to *V*. *fluvialis*, in which only 48% of strains harbor T6SS1 [21], we speculate that T6SS1 may enhance competitive fitness or virulence in *V*. *furnissii*. In addition, all 30 *V*. *furnissii* strains carried the *vfh* gene, underscoring its high conservation. In contrast, *zot* was detected in only five strains (VFN3, C1, PV10, PV88, S0821) (Figure 1), suggesting its potential acquisition through horizontal gene transfer. The functional role of zot in *V*. *furnissii* warrants further experimental investigation.

**Figure 2:**
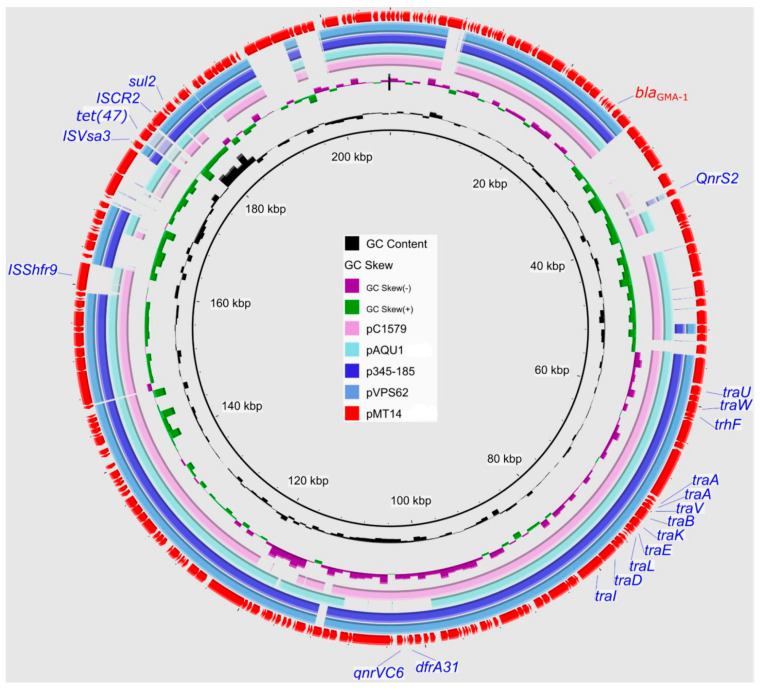
A circular comparison of the pAQU-type plasmid pMT4 with related plasmids from the NCBI database, namely pC1579, pAQU1, p345-185, and pVPS62. The outermost circle denotes pMT14, with arrows representing its coding genes.

The plasmid pMT14 (207, 270 bp) was found to carry six antibiotic resistance genes, *qnrS2*, *qnrVC6*, *dfrA31*, *tetA*, *sul2,* and *bla*_GMA-1_. No known plasmid replicon type could be identified. BLAST (https://blast.ncbi.nlm.nih.gov/Blast.cgi) analysis revealed high similarity to pAQU-type plasmids, with pMT14 uniquely carrying both *qnrS2* and *qnrVC6* (Figure 2). To our knowledge, this is the first report of a pAQU-type plasmid in *V*. *furnissii*. Moreover, genomic analysis revealed that only four out of the thirty *V. furnissii* strains carried plasmids harboring antibiotic resistance genes, specifically pMT14, p104486766-qnrVF1, pVFN3-blaOXA-193K, and a plasmid-origin contig from strain GD23SC5431TM. Notably, an IncA/C2-type replicon was identified in only three of these four resistance-bearing plasmids (Figure 1), underscoring the limited spread of plasmid-mediated resistance in *V*. *furnissii*.

Strain MT14 exhibited resistance to amoxicillin but carried no known penicillin resistance genes other than *bla*_GMA-1_ (Table 1). Since the NCBI database contains sequences for *bla*_GMA_ variants (*bla*_GMA-2_, *bla*_GMA-3_, *bla*_GMA-4_), we therefore synthesized and cloned these genes into the pET28a vector for antimicrobial susceptibility testing. All variants conferred resistance to penicillins (ampicillin, amoxicillin, carbenicillin) but not to cephalosporins, confirming their preferential hydrolysis of penicillin-class antibiotics. In addition, chromosome I of MT14 was found to carry a transposon harboring multiple resistance genes, including *tet*(A), *floR*, and *sul2*. This mobile genetic element was flanked by *Tn3* and integrated into a *umuC*-like gene, suggesting that *umuC*-like genes may serve as genomic hotspots for the insertion of mobile elements, as previously observed in other bacteria species [22].

## 4. Discussion

Our study provides the first evidence that *V*. *furnissii* can acquire and maintain pAQU-type plasmids, representing a previously unrecognized environmental reservoir for these mobile genetic elements. Of particular concern is the resistance profile of strain MT14 to penicillins and ciprofloxacin, which are clinically relevant drugs used to control *Vibrio* infections. More notably, we observed that *V*. *furnissii* strain VFN3 carries the virulence-associated gene *zot* alongside the clinically relevant resistance genes *mph*(A) and *bla*_OXA-1_, while strain GD23SC5431TM harbors *zot*, *bla*_NDM-1_, and *bla*_OXA-1_. The convergence of these virulence and resistance determinants in *V. furnissii* suggests it is an emerging pathogen of clinical concern. Although some resistance genes are located on plasmids, their transferability can vary. For example, p104486766-qnrVF1, hosted by *V. furnissii* 104486766, is conjugative [14], while pMT14 is not. Furthermore, the observed high MIC of ciprofloxacin in strain MT14 is likely attributable to chromosomal mutations in *gyrA* and/or *parC*, a common mechanism for fluoroquinolone resistance. We hypothesize that antibiotic selection pressure in aquaculture may not only enrich for such resistant clones but also facilitate the dissemination of mobile genetic elements. Together, these mechanisms represent a potential public health threat that warrants further surveillance of filter-feeding shellfish in aquaculture.

Our functional characterization confirmed that *bla*_GMA-2_, *bla*_GMA-3_, and *bla*_GMA-4_, like *bla*_GMA-1_, all confer resistance to penicillin-class antibiotics, suggesting that these *bla*_GMA_ variants are penicillin hydrolases. Given that all known *bla*_GMA_ genes have been identified in *Vibrio* species, it is temptation to speculate that *Vibrios* are the original hosts of this gene family. In addition, the fact that most *V. furnissii* strains (73.3%, 22/30; Figure 1) lack resistance genes implies that these traits are not innate but have emerged in some strains, likely captured through plasmid transfer or chromosomal mobile elements.

Although *V. furnissii* is currently considered as an opportunistic human pathogen, several lines of evidence suggest its pathogenic potential may be underestimated. First, the hemolysin gene *vfh*, which contributes to the pathogenicity of the closely related *V. fluvialis* [21], is conserved across all examined *V. furnissii* strains. Second, the T6SS, which plays important roles in bacterial competition and in eukaryotic cell infecting in other *Vibrio* species [17,18], is also widespread in this species. Thirdly, the emergence of *zot*, a toxin known to disrupt the actin cytoskeleton in host cells during infection by both *V. cholerae* and *V. parahaemolyticus* [19,20], could further enhance the virulence of *V. furnissii*. Further research should explore the roles of *vfh*, *zot,* and T6SSs in *V. furnissii* pathogenesis and determine whether this bacterium employs different virulence strategies to infect humans and shellfish.

In conclusion, our findings suggest that *V. furnissii* is not merely an environmental marine bacterium but a neglected reservoir for the dissemination of antibiotic resistance and virulence genes. Therefore, it is critical to enhance the genomic surveillance of drug resistance and virulence gene dissemination in environmental *Vibrios* and to conduct functional studies to elucidate the pathogenicity mechanisms of *V*. *furnissii*.

## Figures and Tables

**Table 1:** Minimum inhibitory concentrations (MICs) for *V. furnissii* strain MT14 and *E. coli* strains.

Strain	Species	MIC (μg/mL)													
		AMP	AMX	CAR	PIP	CED	CXM	CTX	IMP	CIP	KAN	CHL	STR	TET	COL
MT14	*V. furnissii*	>128	>128	>128	>128	8	4	0.5	2	64	16	>128	64	64	0.25
BL21(DE3)-GMA-1	*E. coli*	>128	>128	>128	>128	8	1	0.25	-	-	-	-	-	-	
BL21(DE3)-GMA-2	*E. coli*	128	>128	>128	16	8	2	≤0.25	-	-	-	-	-	-	
BL21(DE3)-GMA-3	*E. coli*	>128	>128	>128	>128	8	2	≤0.25	-	-	-	-	-	-	
BL21(DE3)-GMA-4	*E. coli*	>128	>128	>128	>128	8	1	≤0.25	-	-	-	-	-	-	
ATCC25922	*E. coli*	2	4	2	1	8	4	0.25	0.25	0.25	2	≤0.5	0.5	≤0.25	≤0.25
BL21(DE3)-pET28a	*E. coli*	0.5	2	1	0.5	8	1	≤0.25	-	-	-	-	-	-	

Note: The tested *E. coli* BL21(DE3) strains harbored the empty pET28a vector (control) or the same vector carrying the following genes: GMA-1, GMA-2 (RefSeq: WP_150896653.1), GMA-3 (RefSeq: WP_236110802.1), and GMA-4 (RefSeq: WP_038227092.1). *E. coli* ATCC 25922 was included as a quality control strain. -, not determined. AMP, ampicillin; AMX, amoxicillin; CAR, Carbenicillin; PIP, piperacillin; CED, cephradine; CXM, cefuroxime sodium; CTX, ceftiofur sodium; IMP, imipenem; CIP, ciprofloxacin; CHL, chloramphenicol; TET, tetracycline; KAN, kanamycin; STR, streptomycin; COL, colistin.

## Data Availability

The original data presented in the study are openly available in NCBI database. The complete sequences of strain MT14 were submitted to NCBI database with the accession numbers CP115188-CP115190.
